# Nanotechnology-driven synergy in cardio-oncology: enhancing tumor suppression and reducing cardiotoxicity

**DOI:** 10.3389/fphar.2025.1641618

**Published:** 2025-10-03

**Authors:** Luyao Ma, Bowen Zhang, Xiaomei Liu, Shengwei Gao, Shengjie Kong, Yanfen Li, Ruihua Wang, Meifeng Li, Xinyu Mao, Yuhong Li, Yang Luo, Liang Li, Chunxiao Lv, Yuhong Huang

**Affiliations:** ^1^ Department of Clinical Pharmacology, Second Affiliated Hospital of Tianjin University of Traditional Chinese Medicine, Tianjin, China; ^2^ Institute of Traditional Chinese Medicine, Tianjin University of Traditional Chinese Medicine, Tianjin, China; ^3^ Department of Oncology, Second Affiliated Hospital of Tianjin University of Traditional Chinese Medicine, Tianjin, China

**Keywords:** cardio-oncology, nanotechnology, chemotherapy, synergy, cancer therapy

## Abstract

The integration of nanotechnology into oncology has profoundly reshaped cancer treatment, enabling drug delivery systems with remarkable precision, enhancing antitumor efficacy, and simultaneously addressing major challenges such as cardiotoxicity, one of the most prevalent and serious adverse effects of conventional chemotherapy. This review systematically examines the dual role of nanotechnology, highlighting its capacity to enhance the therapeutic effectiveness of anticancer treatments while concurrently mitigating cardiotoxic side effects. The discussion centers on a broad spectrum of nanocarrier platforms, such as liposome-based, polymeric nanocarriers, and inorganic nanocarriers organized according to their structural features and therapeutic benefits, thereby enabling a systematic comparison with conventional drug delivery strategies. By improving drug bioavailability, enabling controlled release, and achieving precise tumor-specific targeting, these nanocarrier systems enhance antitumor efficacy while concurrently reducing collateral damage to healthy tissues. Moreover, recent preclinical and clinical studies were summarized to demonstrate substantial advances in this interdisciplinary field, while also identifying persistent challenges that remain to be addressed. Finally, the review explores future directions, with particular emphasis on the integration of artificial intelligence to optimize nanocarrier design and the promise of personalized nanomedicine in transforming cancer care. Overall, this work provides a critical foundation for advancing next-generation, patient-tailored cancer therapies.

## 1 Introduction

Cancer continues to represent the leading cause of disease-related morbidity and mortality worldwide. By 2022, approximately 20 million new cancer cases were diagnosed globally, nearly seven million more than in 2020, underscoring the accelerating global cancer burden (Sung et al., 2021; Bray et al., 2024). Although substantial progress has been achieved, illustrated by a 15% increase in the 5-year survival rate of patients in China over the past decade and a half, the unintended consequences of treatment are becoming increasingly evident, raising serious concerns regarding the long-term survivorship of cancer patients (Siegel et al., 2024). Within these complications, cardiotoxicity has emerged as one of the most pressing challenges in oncology. Recent evidence suggests that over 40% of patients receiving chemotherapy experience cardiotoxic effects, making chemotherapy-induced cardiotoxicity (CIC) not a transient complication but a critical determinant of long-term quality of life (López-Sendón et al., 2020; Christidi and Brunham, 2021; Kong et al., 2022). Mechanistically, CIC arises from reactive oxygen species–induced mitochondrial injury, calcium dysregulation, and ferroptosis, manifesting clinically in a spectrum of conditions from arrhythmias to overt heart failure (Tai et al., 2023). This dual challenge, namely, sustaining durable tumor control while simultaneously protecting the cardiovascular system, underscores the urgent need for innovative therapeutic strategies that can preserve oncological efficacy while safeguarding cardiac health (Chen et al., 2022; Su et al., 2022; Cejas et al., 2024).

Nanotechnology provides a transformative strategy for drug delivery by overcoming many of the inherent limitations of conventional chemotherapy. Distinct from traditional nanomedicine that emphasizes tumor targeting alone, cancer nanocardiology advances a dual-functional paradigm that integrates tumor suppression with cardio-protection within a single nanoplatform (Lu et al., 2024)**.** By encapsulating chemotherapeutic drugs, nanocarrier systems enhance solubility (Zeng et al., 2023), enhancing stability (Du et al., 2024), and increasing bioavailability (Itoo et al., 2024). By optimizing pharmacokinetic profiles, these systems enable tumor-specific delivery while minimizing off-target exposure (Lee et al., 2021; Wang et al., 2021; Zhu et al., 2023). Various nanocarriers, including liposomes (Su et al., 2022), polymeric nanocarriers (Feng et al., 2022), dendrimers (Dey et al., 2022), and inorganic nanomaterials (Pei et al., 2023) have demonstrated strong potential for precise drug delivery. Many of these systems can be engineered to achieve stimuli-responsive release triggered by pH, temperature, or enzymatic changes within the tumor microenvironment (Zhang J. et al., 2023). In addition, functionalization with targeting ligands or monoclonal antibodies further improves tumor specificity, markedly reducing the risk of cardiotoxicity (Su et al., 2022; Nevins et al., 2024).

Thus, the objective of this review is to offer a comprehensive synthesis of current applications of nanotechnology in cancer therapy, with particular emphasis on its capacity to improve therapeutic efficacy while simultaneously mitigating cardiotoxic side effects. It further examines recent advances in nanocarrier design and evaluates their translational potential across both preclinical and clinical settings. By synthesizing these innovations, the review seeks to elucidate the ways in which nanotechnology may reshape conventional cancer treatment paradigms and ultimately facilitate the development of safer and more effective therapeutic strategies.

## 2 Types and functions of nanocarriers

**FIGURE 1:**
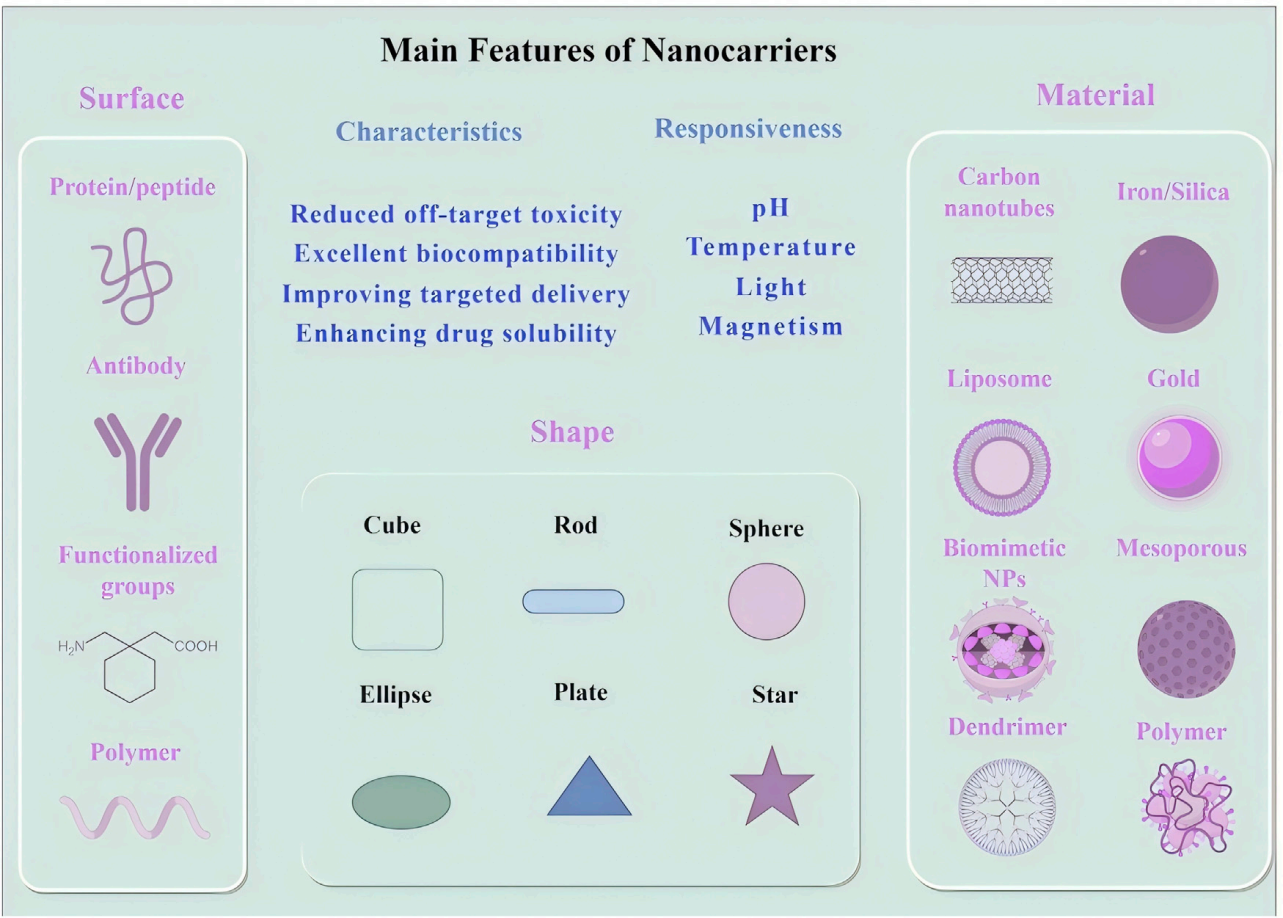
Main feature of different types nanocarriers.

Nanotechnology has introduced innovative drug delivery strategies that are transforming cancer treatment by improving drug stability, solubility, and bioavailability. A diverse range of nanocarriers, including liposomes, polymeric nanocarriers, inorganic nanocarriers, and carbon-based materials, have been developed to enhance the precision and efficiency of oncological drug delivery (Garbayo et al., 2020; Vazhappilly et al., 2021) (Table 1 summarizes the characteristics of different nanocarrier types). Among these, liposomes constitute one of the earliest and most extensively utilized nanocarrier systems. Liposomes, composed of a phospholipid bilayer, can encapsulate both hydrophilic and hydrophobic agents. Their intrinsic ability to fuse with cellular membranes enables direct transport of therapeutic agents into tumor cells (Zhou et al., 2016; Vazhappilly et al., 2021). Polymeric nanocarriers, often synthesized from biodegradable polymers such as poly (lactic-co-glycolic acid) (PLGA), enable controlled drug release at tumor sites, thereby maintaining therapeutic concentrations while reducing systemic toxicity (Lin et al., 2023; Beach et al., 2024). Inorganic nanocarriers, such as gold-based or silica-based systems, exhibit unique physicochemical properties that enable their application in both therapeutic and diagnostic modalities (theranostics). For example, gold nanocarriers are particularly effective in photothermal therapy (Yang et al., 2020; Miao et al., 2022; Hirschbiegel et al., 2023). Carbon-based nanocarriers, such as carbon nanotubes and fullerenes, are structurally robust and capable of penetrating dense tissue matrices, thereby facilitating drug delivery into deep-seated tumors (Tang et al., 2021; Kaurav et al., 2023). Moreover, nanocarriers derived from natural biomaterials, such as protein-based nanocarriers and virus-like nanocarriers (VLPs), mimic viral architectures to promote cellular uptake. These carriers are biodegradable and display low immunogenicity, rendering them promising candidates for clinical translation (Zhang et al., 2017; Habibi et al., 2022; Tenchov et al., 2022) (Figure 1 illustrates the principal features of different nanocarrier types).

**TABLE 1:** Relevant characteristics of different types of nanocarriers.

Nanocarrier	Type	Size (nm)	Drug-carrying capacity	Biocompatibility	Degradation pathways	Advantages	Disadvantages	References
Lipid-based carriers	Liposomes	50–200	High	Excellent	Biodegradable (phospholipids)	Prominent controlled drug release, Rich surface modification	Limited stability in circulation	Guimarães et al. (2021), Kurano et al. (2022)
	Solid Lipid Nanocarriers	50–500	Moderate	Good	Biodegradable	High stability, controlled release	Restricted biodistribution	Scioli Montoto et al. (2020), Sivadasan et al. (2023)
	Nanostructured Lipid Carriers	50–1000	High	Excellent	Biodegradable	High drug loading, suitable for various drugs	Difficulty in production	Syed Azhar et al. (2022)
Polymer-based carriers	Polymeric Nanocarriers	10–1000	Moderate to High	Good	Hydrolysis or Enzymatic Degradation	High drug loading, Controlled release, Biocompatibility	Complex production, Restricted biodistribution, Limited stability	Xu et al. (2020), Sivadasan et al. (2023)
	Polymeric Micelles	10–100	Moderate	Good	Biodegradable	High solubility and strong targeting capability	Limited stability in circulation	Ghezzi et al. (2021), Ghosh and Biswas (2021)
	Dendrimers	1–10	High	Good	Hydrolysis or Enzymatic Degradation	Prominent controlled drug release, High drug-carrying capacity	Complex preparation, Potential toxicity	Kaup and Velders (2022), Phatale et al. (2022)
	Nanomicelles	10–100	Moderate	Good	Hydrolysis or Enzymatic Degradation	High solubility and strong targeting capability, Excellent biocompatibility	Limited stability, Complex formulation, Potential toxicity	Barani et al. (2021), Li et al. (2022)
Inorganic carriers	Metal Nanocarriers	1–100	Variable	Varies	Non-degradation or slow degradation	Strong optical properties, High reactivity, Versatile applications	Potential for long-term toxicity	Saifi et al. (2021)
	Quantum Dots	2–10	Low	Varies	Non-degradation or slow degradation	Excellent optical performance for imaging	Poor surface modification, Potential toxicity	Lin and Chen (2023), Li et al. (2024a)
	Nanoshells	10–200	High	Good	Non-degradation or slow degradation	Tunable properties, Enhanced imaging, Efficient drug delivery	Complex synthesis, High cost, Potential toxicity	Zhao et al. (2023), Kim et al. (2024)
	Silica Nanocarriers	10–200	High	Good	Non-degradation or slow degradation	High biocompatibility, Easy surface modification, Low toxicity	Limited biodegradability, Potential aggregation, Complex functionalization	Huang et al. (2022), Chithra et al. (2025)
	Iron Oxide Nanocarriers	10–100	Moderate	Good	Redox Reaction	Magnetic properties, Biocompatibility, Easy surface modification	Potential toxicity, Aggregation, Limited stability	Wu et al. (2022), Araújo et al. (2024), Kumar et al. (2024)
Carbon-based carriers	Carbon Nanotubes	1–50	High	Varies	Oxidation Reaction and Enzyme	High strength, Electrical conductivity, Thermal stability	Potential toxicity, Difficult dispersion, Complex production	Jin et al. (2022), Tang et al. (2022)
	Graphene and its Derivatives	1–100	High	Fair	Difficult to Degrade	High conductivity, Mechanical strength, Versatile applications	Potential toxicity, Production challenges, Aggregation issues	Quan et al. (2017), Singh et al. (2022)
	Fullerenes	0.7–1.5	Moderate	Good	Non-Degradation	High electron affinity, Photostability, Versatile chemical reactivity	Production cost, Limited solubility, Potential toxicity	Wang and Zhan (2021), Bolshakova et al. (2025)
bio-based carrier	Protein Nanocarriers	10–200	Moderate to High	Excellent	Enzymatic Degradation	Biocompatibility, Targeted delivery, Biodegradability	Limited stability, Complex production, Short shelf life	Kianfar (2021), Nguyen et al. (2023)
	Polysaccharide Nanocarriers	10–200	Moderate	Good	Enzymatic Degradation	Biocompatibility, Biodegradability, Low toxicity	Limited stability, Complex formulation	Swierczewska et al. (2016), Allawadhi et al. (2022)
	Virus-like nanocarriers	20–200	Moderate	Excellent	Biodegradable	High immunogenicity, Safety (non-replicating), Versatile applications	Complex production, Potential instability, Costly manufacturing	Chen et al. (2023), Sun et al. (2024)

To further improve specificity, targeting ligands or monoclonal antibodies may be conjugated to the surface of nanocarriers, thereby enabling active targeting of tumor tissues while sparing healthy organs, especially the heart. This targeted approach is particularly valuable for stimulus-responsive drug release triggered by specific cues within the tumor microenvironment, such as alterations in pH or temperature, thereby maximizing therapeutic efficacy while minimizing off-target toxicity.

## 3 The role of nanotechnology in reducing cardiotoxicity in antitumor therapy

Nanotechnology enhances the efficacy of antitumor therapies by enabling targeted drug delivery, controlled release, and multimodal treatment strategies, thereby overcoming many limitations associated with conventional regimens.

### 3.1 Pathophysiology of cardiotoxicity induced by antitumor therapy

**FIGURE 2:**
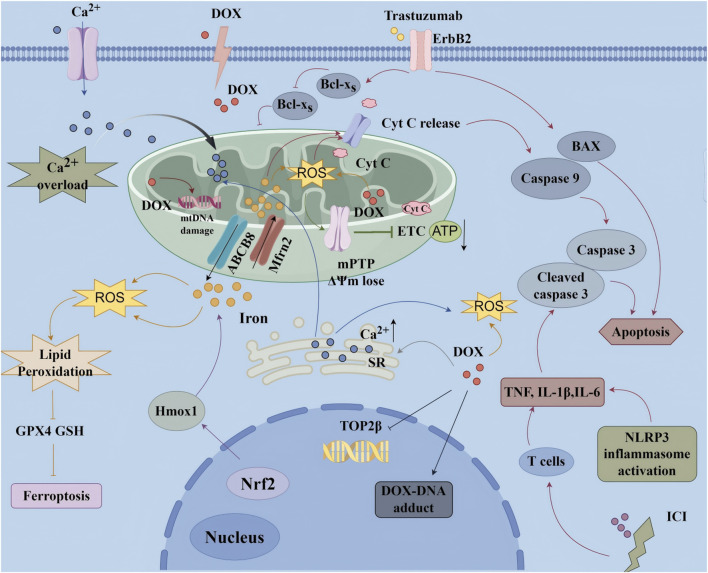
Molecular mechanism of chemotherapy-induced cardiotoxicity ATP, Adenosine Triphosphate; BAX, Bcl-2 Associated X Protein; Cyt C, Cytochrome C; DOX, Doxorubicin; ΔΨm, Mitochondrial Membrane Potential; ErbB2, Epidermal Growth Factor Receptor 2; ETC., Electron Transport Chain; GPX4, Glutathione Peroxidase 4; GSH, Glutathione; ICI, Immune Checkpoint Inhibitors; IL-1β, Interleukin-1 beta; IL-6, Interleukin-6; mPTP, Mitochondrial Permeability Transition Pore; Nrf2, Nuclear Factor Erythroid 2-Related Factor 2; ROS, Reactive Oxygen Species; TNF-α, Tumor Necrosis Factor-alpha.

CIC encompasses a wide range of structural and functional cardiac complications-most notably heart failure (HF), arrhythmias, myocardial ischemia, and coronary artery disease (Carrasco et al., 2021; Li et al., 2021). These complications are frequently severe and potentially life-threatening, with HF representing the most critical clinical manifestation. Major contributors include anthracyclines (e.g., doxorubicin, DOX), targeted therapies (e.g., trastuzumab), and immune checkpoint inhibitors (Zhang et al., 2015). The pathophysiology is multifactorial, characterized by oxidative stress, mitochondrial dysfunction, and impaired cardiomyocyte signaling (Yang et al., 2023), as shown in Figure 2. DOX, for example, drives excessive ROS generation that results in DNA damage, lipid peroxidation, apoptosis or necrosis, and severe disruption of mitochondrial energy metabolism (Ding et al., 2023). Trastuzumab, in contrast, disrupts mitochondrial biogenesis and function through ErbB2 inhibition, thereby suppressing essential survival pathways and precipitating contractile dysfunction (Ye et al., 2023).

Recent studies have highlighted ferroptosis as a central mechanism contributing to CIC. DOX, along with agents such as cisplatin and sorafenib, disrupts iron homeostasis, suppresses GPX4 and GSH, and activates ACSL4, collectively leading to iron overload, lipid peroxidation, and cardiomyocyte ferroptosis (Ding et al., 2023). Trastuzumab-induced activation of SLC7A11 appears to further sensitize cardiomyocytes to ferroptotic death (Zhang et al., 2023). Inflammation emerges as another critical factor: anticancer agents activate cardiac macrophages and recruit circulating monocytes, neutrophils, and T cells, which in turn release TNF-α, IL-1β, IL-6, chemokines, and reactive species, thereby exacerbating cardiomyocyte injury, fibrosis, and adverse remodeling. Notably, immune checkpoint inhibitors may provoke autoimmune-like myocarditis characterized by extensive T-cell infiltration (Wei et al., 2021; Zhao et al., 2025).

Another hallmark of CIC is the disruption of Ca^2+^ homeostasis. Anthracyclines and related agents impair SR Ca^2+^ reuptake through SERCA2a dysfunction and promote Ca^2+^ leakage via RyR2 channels, thereby inducing cytosolic Ca^2+^ overload. This disruption interferes with excitation-contraction coupling, facilitates arrhythmogenesis, and provokes ER stress with subsequent UPR activation, ultimately culminating in apoptosis (Ayza et al., 2020; Wei et al., 2021; Dridi et al., 2023; Li W. et al., 2024). Although cardioprotective strategies such as dexrazoxane, β-blockers, and ACEi/ARB have been developed, CIC persists as a formidable clinical challenge. Its multifaceted mechanisms limit optimal oncologic dosing and regimens, while also compromising long-term survivorship (Wei et al., 2021). These challenges underscore the urgent need for mechanism-driven innovations-such as rationally designed nanomaterials-that can selectively target ferroptosis and inflammation without undermining antitumor efficacy.

### 3.2 Applications of nanotechnology in reducing cardiotoxicity

Nanocarriers, including liposomes and polymeric or inorganic nanocarriers, significantly mitigate chemotherapeutic cardiotoxicity through selective drug delivery. These carriers reduce nonspecific drug accumulation in myocardial tissue by means of physical optimization and surface modification with targeting ligands (Zhao et al., 2020; Vazhappilly et al., 2021). This approach represents an innovative strategy for cardio-protection during chemotherapy (Garbayo et al., 2020).

#### 3.2.1 Promoting targeted drug delivery

**FIGURE 3:**
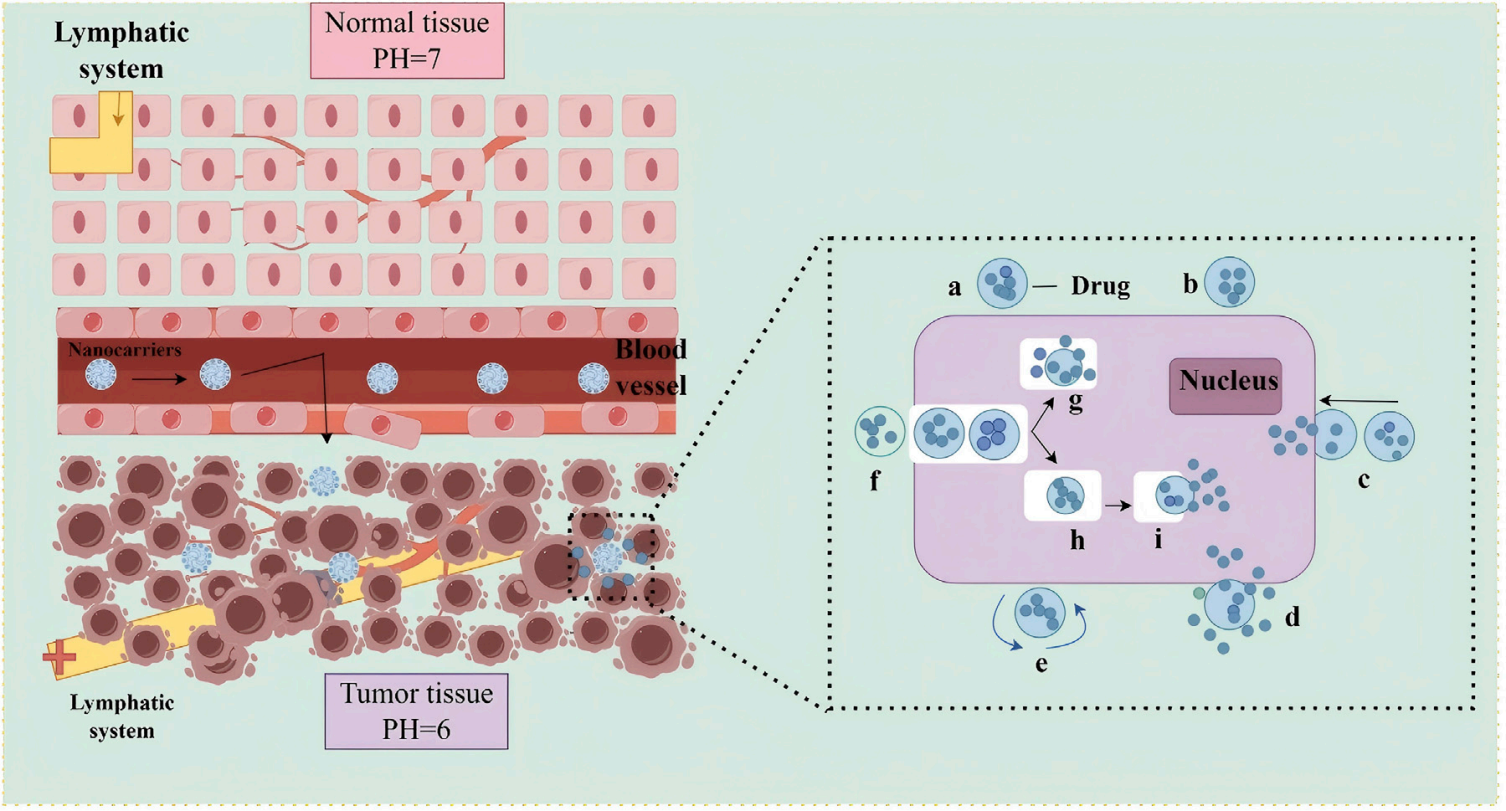
Schematic illustration of passive targeting drug delivery using nanocarriers. Left: Microvascular permeability comparison between normal and tumor tissues. In normal tissues (top), intact endothelial junctions and functional lymphatic drainage prevent nanonanocarrier extravasation. In tumor tissues (bottom), structurally abnormal vasculature with enlarged endothelial gaps (red arrows) and impaired lymphatic system (yellow cross) enable preferential nanonanocarrier accumulation *via* the EPR effect, facilitated by the acidic tumor microenvironment (pH 6.0 vs normal pH 7.0). Right: Intracellular delivery mechanism of nanocarriers, including: (a) Drug encapsulation in nanocarriers; (b) Surface receptor binding; (c) Endocytosis by tumor cells; (e) Endosomal escape; (g) Intracellular drug release; (h) Drug translocation to intracellular targets (e.g., nucleus). Abbreviation: EPR, Enhanced Permeability and Retention.

Nanocarriers employ both passive and active targeting mechanisms. Passive targeting is mediated by the enhanced permeability and retention (EPR) effect, which allows nanocarriers of 10–200 nm in size to preferentially accumulate in tumor tissues due to their leaky vasculature (Allawadhi et al., 2022; Singh et al., 2022; Chen et al., 2023; Sun et al., 2024), as shown in Figure 3. This selective distribution reduces systemic exposure, minimizes off-target toxicity, and enhances therapeutic efficacy. For instance, stimuli-responsive nanocarriers have been designed to release their drug payload in response to the acidic tumor microenvironment. These carriers remain stable under physiological pH (7.0) but release drugs efficiently at lower pH (5.0–6.5) (Liu et al., 2014). A dextran–DOX conjugate, for example, released only 11% of its payload at pH 7.4, compared to 96% at pH 4.0 (Behera and Padhi, 2022). This controlled release improves drug efficacy at tumor sites while protecting healthy tissues, including cardiomyocytes (Zhang et al., 2020; Yu et al., 2021).

**FIGURE 4:**
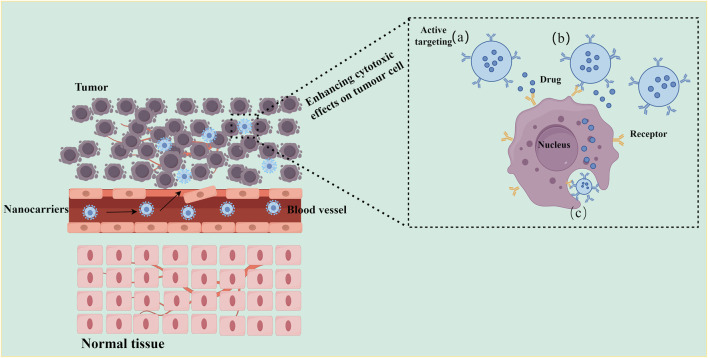
Schematic illustration of active targeting drug delivery using nanocarriers. Left: Schematic showing nanonanocarrier extravasation and tumor targeting. Nanocarriers modified with target ligands (blue stars) circulate through blood vessels, cross the abnormal tumor vasculature (dashed red arrows), and specifically bind to tumor cell surface receptors (orange Y-shaped structures), while being excluded from normal tissue (bottom) due to lack of target receptors. Right: Three active targeting delivery modes: **(a)** Proximity release: nanocarriers release drugs in the tumor microenvironment upon receptor binding; **(b)** Membrane depot: Ligand-receptor interaction anchors nanocarriers to the cell membrane for sustained drug release; **(c)** Receptor-mediated endocytosis: nanocarriers are internalized into tumor cells, delivering drugs directly to intracellular targets (e.g., nucleus). Key feature: Active targeting relies on specific ligand-receptor interactions (e.g., antibody-antigen, peptide-receptor), enabling selective drug accumulation in tumor tissues while minimizing uptake by normal cells.

The EPR effect, however, can be inconsistent due to intertumoral heterogeneity (Fang et al., 2020; Irannejadrankouhi et al., 2025). Active targeting strategies have therefore been developed to improve reliability. These involve functionalizing nanocarriers with surface ligands such as antibodies or aptamers that recognize receptors overexpressed on tumor cells (Mi et al., 2020; Wang et al., 2023). This approach enhances specificity and facilitates intracellular drug delivery, as shown in Figure 4. Clinical evidence shows that liposomal DOX reduces the risk of cardiotoxicity by 54% compared to conventional DOX (OR = 0.46, p = 0.03) and is associated with a smaller decline in left ventricular ejection fraction (2.1% vs 5.6%, p = 0.0014) (Rayson et al., 2012; Xing et al., 2015). In HER2-positive breast cancer, trastuzumab-modified nanocarriers lowered the incidence of cardiac complications to 2.4% while enhancing therapeutic outcomes (Ngamcherdtrakul et al., 2015; Meng et al., 2018).

**FIGURE 5:**
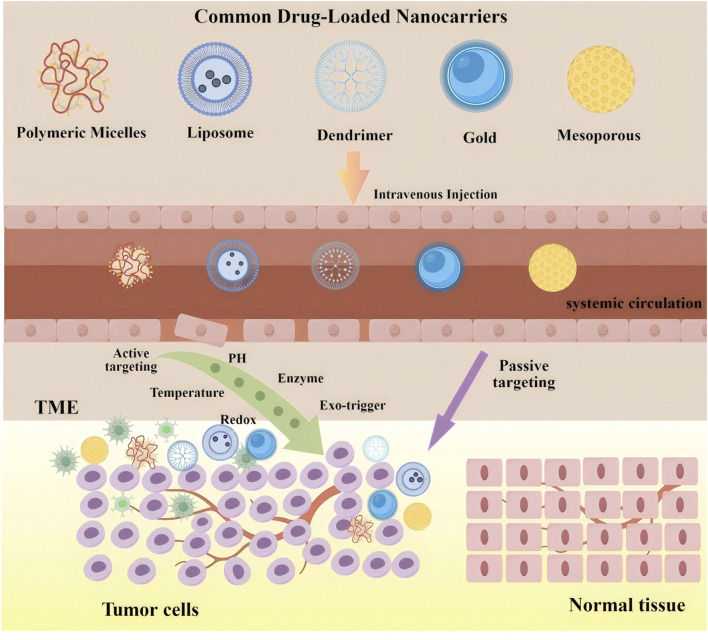
The process of nanocarriers as drug carriers acting on cancer cells. The upper shows structural diversity of common drug-loaded nanocarriers (left to right: polymeric micelles, liposomes, dendrimers, gold nanocarriers, mesoporous nanocarriers) as versatile drug encapsulation platforms. The lower depicts three-stage *in vivo* delivery: (1) Systemic circulation-intravenous injection enables bloodstream entry and systemic distribution; (2) tumor targeting *via* dual mechanisms: passive targeting (purple arrow, EPR effect-mediated extravasation across disorganized tumor vasculature) and active targeting [green arrow, triggered by TME cues (pH, enzymes, redox) or exogenous stimuli (temperature, light)]; and (3) Intratumoral action-drug release within the tumor microenvironment (TME) for selective cancer cell interaction while sparing normal tissues. Critical to this process, TME-established pathophysiological gradients (e.g., acidic pH, elevated enzyme levels) provide spatiotemporal control signals for stimuli-responsive nanocarriers. This multi-stage paradigm integrates dual targeting to overcome biological barriers, enhancing cancer therapeutic index.

By combining passive targeting through the EPR effect with active targeting via ligand modification, dual-targeting strategies significantly improve the therapeutic index of anticancer drugs (Izci et al., 2021). This dual approach also enables deeper penetration into the tumor microenvironment—a site where many conventional therapies fail due to inadequate drug diffusion. Figure 5 illustrates the process by which nanocarriers act as drug delivery vehicles within cancer cells.

#### 3.2.2 Multifunctional nanocarriers for cardioprotection

Multifunctional nanocarriers integrate therapeutic and diagnostic functions by simultaneously enabling multi-drug co-delivery, controlled release, and synergistic effects. These platforms co-deliver chemotherapeutic agents, immunomodulators, and cardioprotectants such as coenzyme Q10, cardioprotective peptides, and natural bioactive compounds derived from Traditional Chinese Medicine (TCM) (e.g., resveratrol, quercetin, curcumin, berberine), which possess potent antioxidant and anti-inflammatory properties (Sun et al., 2025). In this way, they achieve both tumor suppression and organ protection (Majumder and Minko, 2021; Long et al., 2024). Mechanistically, multifunctional systems provide three major advantages. First, temporal release control coordinates the kinetics of drug and cardioprotectant delivery, preserving antitumor efficacy while reducing cardiotoxicity (Carvalho et al., 2021); Second, ROS-scavenging functions mediated by superoxide dismutase (SOD) mimetics protect against chemotherapy-induced oxidative myocardial injury (Quagliariello et al., 2020). Third, theranostic features allow real-time monitoring of treatment response, enabling personalized therapy adjustments (Shetty et al., 2019). Collectively, these advances highlight the potential of multifunctional nanocarriers as platforms for overcoming tumor drug resistance while simultaneously protecting cardiac function.

#### 3.2.3 Distinct advantages of nano-cardio-oncology

**FIGURE 6:**
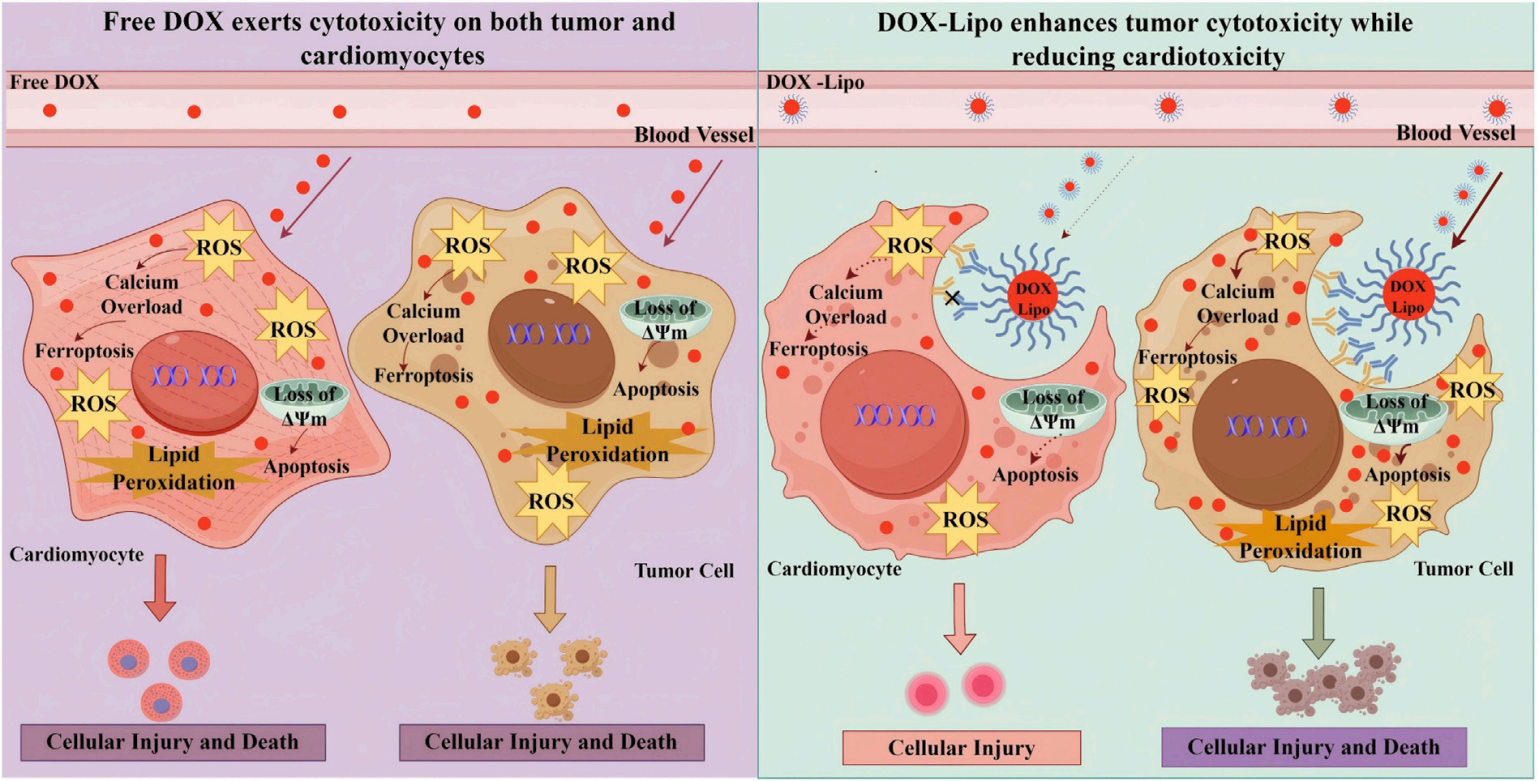
Pathological Mechanisms of Free DOX vs DOX-Lipo in Cardiomyocytes and tumor Cells. In DOX therapy, the use of free DOX often results in simultaneous damage to both cardiomyocytes and tumor cells due to its lack of selectivity, leading to significant cardiac toxicity and adverse effects. On the other hand, DOX-Lipo enhances the drug’s targeting ability, promoting tumor cell apoptosis and death while reducing cardiac toxicity. DOX,Doxorubicin; DOX-DNA adduct,Doxorubicin-DNA adduct; DOX-Lipo, Doxorubicin Liposome; ΔΨm, Mitochondrial Membrane Potential; TOP2β, Topoisomerase 2 beta.

Cardio-oncology nanocarriers establish a unique therapeutic paradigm that combines anticancer efficacy with cardio-protection, distinguishing them from conventional nanomedicine, which primarily focuses on tumor targeting and drug delivery efficiency. Poly (methacrylate citric acid)/DOX nanocarriers, for example, demonstrate 1.5-fold greater antitumor efficacy compared with free DOX in preclinical models, while simultaneously reducing systemic and cardiotoxicity (Yu et al., 2022). As shown in Figure 6, these nanocarriers must fulfill three critical requirements: they should achieve high tumor accumulation, enable effective cardio-protectant release in cardiac tissue, and prevent cross-interference between therapeutic components. Cascading-responsive nano-systems exemplify this concept by modulating drug release kinetics according to the distinct biological characteristics of tumor and cardiac microenvironments (Huang et al., 2025).

Furthermore, cardio-oncology nanomedicine integrates advanced multidisciplinary approaches. Cardiovascular molecular imaging allows real-time monitoring of cardiac function; computational modeling predicts drug-induced cardiotoxicity; and organ-on-a-chip platforms simulate interactions between the heart and tumor tissues. Clinically, this discipline has opened transformative pathways, with several agents progressing through clinical trials. Notably, liposomal DOX formulations demonstrated more than 60% reduction in cardiac adverse events in Phase II trials (Moskowitz et al., 2021). Collectively, these innovations establish cardio-oncology nanomedicine as a distinct research ecosystem. By providing standardized frameworks and emphasizing the integration of therapy and protection, nanoplatform-based cardio-oncology is emerging as a new standard in comprehensive cancer care.

## 4 Preclinical and clinical research progress

### 4.1 Preclinical research

Lipid-based nanocarriers have emerged as effective drug delivery platforms due to their excellent biocompatibility and their ability to encapsulate both hydrophilic and hydrophobic compounds (Zhou et al., 2016). Liposomal formulations, such as liposomal DOX, are less cardiotoxic while maintaining strong antitumor efficacy. In preclinical studies, DOX-loaded liposomes administered to mice with triple-negative breast cancer reduced tumor growth by more than 50% while causing minimal cardiac injury, underscoring their therapeutic potential and the role of macrophage targeting (Lu et al., 2023). Similarly, polymeric micellar nanocarriers carrying paclitaxel reduced systemic toxicity, particularly in cardiac tissue, and improved survival in rat models of cancer-induced cardiotoxicity.

Polymeric nanocarriers further enhance therapeutic precision through controlled drug release and functionalization (Ahmed et al., 2021). For example, folate-targeted liposomes co-delivering paclitaxel and vinorelbine improved tumor suppression in non-small cell lung cancer (NSCLC) models while reducing systemic and cardiac damage compared with free drugs (Karpuz et al., 2021). PEG-b-PCL micelles delivering paclitaxel, cyclopamine, and gossypol demonstrated improved tumor control in ovarian cancer models with reduced cardiotoxicity (Cho et al., 2013). Nevertheless, long-term safety requires careful evaluation. Although polycaprolactone (PCL) is biodegradable, its hydrolysis product, ε-caprolactone, may gradually accumulate in cardiac tissues and induce oxidative stress over prolonged exposure, even though this effect was not evident in short-term studies (Inglut et al., 2020). These results indicate that polymeric platforms could expand therapeutic windows and minimize side effects, although long-term risks must be considered.

Inorganic nanocarriers, including gold nanocarriers and mesoporous silica nanocarriers, show considerable promise for imaging and drug delivery (Nam et al., 2013). Magnetic liposomes loaded with DOX significantly reduced breast tumor volume and caused less cardiotoxicity than conventional formulations (Maghsoudi et al., 2023). In thyroid cancer models, selenium nanocarriers combined with pH-responsive fingolimod enhanced drug release at tumor sites, reducing systemic side effects (Zou et al., 2021). In liver cancer, an UiO-66/Bi2S3 nanocomposite enabled controlled DOX release, suppressed tumor growth, and minimized systemic effects, including cardiac complications (Liu et al., 2022). However, preclinical studies also suggest that gold nanocarriers may accumulate in cardiac tissue over time, potentially inducing oxidative stress via Fenton chemistry reactions (Dulf et al., 2024).

Carbon-based nanomaterials, such as carbon nanotubes (CNTs) and graphene oxide, exhibit high drug-loading capacity and improved tissue penetration (Pei et al., 2023). For instance, RGD-conjugated PLGA nanocarriers increased the therapeutic index of cisplatin in lung cancer models by enhancing tumor regression while reducing systemic toxicity, including nephrotoxicity and cardiotoxicity (Yadav et al., 2023). Graphene oxide-based multilayer nanocarriers co-delivering DOX and methotrexate facilitated transdermal drug delivery, promoted tumor regression, and reduced systemic toxicity, including cardiotoxicity (Rajeev et al., 2023). Despite these advantages, carbon-based nanomaterials require careful assessment of long-term safety. While short-term cardiotoxic effects appear minimal, persistent concerns include aspect ratio-dependent toxicity, irreversible aggregation in physiological environments, and variability in large-scale production quality (Rezaei et al., 2025).

Bio-based nanocarriers derived from proteins, peptides, or polysaccharides offer superior biocompatibility and unique opportunities for functionalization (Torrini et al., 2024). For example, albumin-based nanocarriers carrying paclitaxel palmitate achieved high drug-loading efficiency and promoted significant tumor regression in mouse models, improving bioavailability while reducing systemic toxicity (Lan et al., 2023). Likewise, chitosan-coated silver nanocarriers loaded with 5-fluorouracil and nisin reduced tumor burden in skin cancer models and minimized systemic side effects (Rana et al., 2022). These findings highlight the potential of natural biomaterials for safer and more effective drug delivery.

The focus on targeting precision, functionalization, and biocompatibility provides a strong foundation for next-generation nanocarrier-based cancer therapies with improved safety profiles. Nonetheless, preclinical research has inherent limitations. Many studies rely on small sample sizes, which reduce statistical power and generalizability. Rodent models also differ physiologically from humans, limiting the accuracy with which they replicate human cardiotoxicity mechanisms and pharmacokinetics. Moreover, most studies are of short duration and cannot adequately assess long-term cardiac effects. These constraints emphasize the need for cautious interpretation of preclinical results and highlight the challenges of translating findings directly to clinical applications (L’Abbate et al., 2022). Table 2 summarizes key findings from animal studies employing different nanocarrier systems, providing an overview of their therapeutic potential and safety. Collectively, these investigations suggest that nanocarrier-based strategies could enhance anticancer efficacy while reducing cardiac and systemic toxicities.

**TABLE 2:** Summarizes key findings from preclinical studies involving different nanocarrier systems in animal models.

Nanocarrier type	Drug encapsulated	Tumor model	Targeting mechanism	Antitumor efficacy	Cardiotoxicity reduction	References
Lipid-based carriers	DOX	Breast cancer (mice)	Active targeting *via* EPR	Significant tumor regression	Markedly reduced cardiotoxicity	Quagliariello et al. (2020)
	Paclitaxel	Lung cancer (rats)	Active targeting	Enhanced drug accumulation in tumor	Reduced cardiotoxicity	Peixoto et al. (2021)
	Irinotecan	colon cancer (rats)	Enhanced colon targeting	Increased drug concentration in the tumor	Reduced cardiotoxicity	Bhatia et al. (2024)
Polymer-based carriers	Paclitaxel	Lung cancer (mice)	Active (Folate-R) targeting	Tumor inhibition	Minimal cardiac impact	Yang et al. (2022)
	Paclitaxel	Lung cancer (mice)	Active targeting *via* EPR	Significant tumor regression	Reduced cardiotoxicity	Lu et al. (2023)
	paclitaxel, cyclopamine, and gossypol	Ovarian cancer (mice)	Enhanced delivery targeting	Significant tumor regression	limited cardiotoxicity	Bhaskaran et al. (2022)
Inorganic carriers	Cisplatin	Ovarian cancer (mice)	Gold nanonanocarrier-based	Enhanced prolonged drug retention in tumor cells	Not addressed	Karpuz et al. (2021)
	DOX	Breast cancer (mice)	Magnetic targeting	Enhanced tumor suppression	Reduced cardiotoxicity	Maghsoudi et al. (2023)
	Fingolimod	Thyroid cancer (rats)	pH-responsive release targeting acidic tumor microenvironment	enhanced drug accumulation at tumor site	Minimal cardiac impact	Zou et al. (2021)
	DOX	Hepatocellular carcinoma (rats)	Enhanced targeting	Significant tumor regression	Reduced cardiotoxicity	Liu et al. (2022)
Carbon-based carriers	Cisplatin	Lung cancer (rats)	Enhanced delivery targeting	Enhanced tumor inhibition	Lower systemic toxicity, including reduced cardiotoxicity	Yadav et al. (2023)
	DOX and Methotrexate	Breast cancer (rats)	Transdermal delivery system for localized treatment	Enhanced tumor inhibition	Reduced cardiotoxicity	Rajeev et al. (2023)
Bio-based carrier	Paclitaxel	Breast cancer (mice)	Active targeting *via* EPR	Significant tumor regression	Reduced cardiotoxicity	Torrini et al. (2024)
	Gallium-Polyphenol	Lung cancer (mice)	Depleting local lung microbiota	Improved chemotherapy efficacy	Reduced cardiotoxicity	Han et al. (2023)
	5-Fluorouracil and Nisin	Skin cancer (mice)	Active targeting *via* EPR	Significant tumor suppression	Not explicitly reported, but improved drug delivery reduces off-target toxicity	Rana et al. (2022)
	Oxaliplatin	colon cancer (rats)	Enhanced targeting	Enhanced tumor regression	Reduced systemic toxicity	Mirdamadian et al. (2022)

### 4.2 Clinical trial progress

Lipid-based nanocarriers, particularly liposomes, have been extensively investigated due to their biocompatibility and capacity to encapsulate both hydrophilic and hydrophobic agents (Zhou et al., 2016). A meta-analysis demonstrated that pegylated liposomal doxorubicin (PLD) significantly reduced the risk of congestive heart failure compared with other anthracyclines (OR = 0.34, 95% CI: 0.24–0.47) (Rafiyath et al., 2012). Another study reported no significant difference in 3-year disease-free survival between PLD and epirubicin (94.9% vs 95.4%) in the neoadjuvant or adjuvant treatment of breast cancer, although the incidence of cardiotoxicity was markedly lower in the PLD group (Zhang et al., 2021). These findings underscore the clinical advantage of liposomal formulations in reducing cardiac risk without compromising therapeutic efficacy.

Polymeric nanocarriers, including those synthesized from PLGA and PEGylated materials, are particularly attractive due to their sustained drug release and stability in circulation, making them suitable for targeted cancer therapies (Maghsoudi et al., 2020). A Phase I/II clinical trial of CRLX101, a camptothecin-based nanocarrier, showed encouraging outcomes. In combination with bevacizumab, CRLX101 achieved an objective response rate of 21%, a disease control rate of 86%, and a median progression-free survival of 9.9 months in patients with advanced renal cell carcinoma (Keefe et al., 2016). These systems are often engineered for tumor accumulation, thereby reducing systemic toxicity and enhancing therapeutic efficacy (Li X. et al., 2024). Collectively, polymeric nanocarriers represent a promising approach for precise drug delivery, improving tumor targeting while minimizing damage to healthy organs.

The growing body of clinical evidence highlights the potential of nanocarriers to improve cancer treatment outcomes while mitigating cardiotoxicity. Table 3 summarizes key clinical findings, providing an overview of the progress achieved thus far. Nevertheless, translating dual-purpose nanocarrier systems into clinical oncology remains challenging. Barriers include stringent regulatory requirements for therapies with both anticancer and cardioprotective functions, the complexity of evaluating long-term cardiotoxicity, and the technical difficulties of large-scale clinical-grade nanocarrier production (Makwana et al., 2021; Abdellatif et al., 2022; Santin et al., 2023; Sarfraz et al., 2023; Desai et al., 2025). Future research should focus on systematically assessing the long-term safety of nanotechnology platforms, particularly their potential immunological impacts (Moazzam et al., 2024). At the same time, standardized manufacturing protocols and advanced characterization methods are needed to optimize the precision of smart nanocarriers, thereby improving tumor specificity and minimizing off-target effects (Ali et al., 2021). To use nanotechnology to its fullest potential in cancer and heart defense, these kinds of improvements are needed.

**TABLE 3:** Summarizes key findings from clinical studies involving different nanocarriers.

Phase	Nanocarrier type	Tumor type	Sample size(n)	Endpoint	Key findings	Challenges	References
III	Liposomal Nanocarriers (PEG-Dox)	Metastatic Breast Cancer	509	PFS, OS, ORR, CI, QoL, QoL	Pegylated liposomal doxorubicin showed reduced cardiotoxicity compared to conventional doxorubicin without compromising therapeutic efficacy.	Accessibility to newer formulations; increased cost burden for patients.	O’Brien et al. (2004)
II	Polymer-based Nanocarriers	Advanced Renal Cell Carcinoma	114	PFS, ORR, OS, CI	CRLX101 in combination with bevacizumab demonstrated improved efficacy over standard care in advanced renal cell carcinoma.	Further validation required for large-scale clinical adoption; potential issues with nanocarrier clearance and toxicity.	Voss et al. (2017)
I/Ib	Polymer-based Nanocarriers (siRNA)	Various tumor Types	24	DLT, TE, GSE	First-in-human trial of targeted siRNA nanocarrier demonstrated acceptable safety profiles with encouraging preclinical to clinical translatability.	Complexities in siRNA delivery and degradation; large-scale manufacturing hurdles.	Zuckerman et al. (2014)
I/IIa	Polymer-based Nanocarriers	Metastatic Renal Cell Carcinoma	37	MTD, DLT, ORR, PFS	Demonstrated clinical benefit in advanced renal cell carcinoma when combined with bevacizumab.	Managing off-target effects and nanocarrier clearance in human subjects.	Keefe et al. (2016)
I	Gadolinium-based Nanocarriers	Brain Metastases	15	DLT, MTD	AGuIX nanocarriers enhanced radiosensitization, showing improved tumor response rates without significant additional toxicity.	Long-term safety and gadolinium accumulation in the body require further study.	Verry et al. (2021)
I/II	Gadolinium-based Nanocarriers	Brain Metastases	15	DLT, MTD, Adverse Event	MRI imaging demonstrated precise quantification of nanocarrier uptake in brain metastases, aiding in therapy personalization.	Requires advanced imaging technology and standardization of uptake measurement protocols.	Bennett et al. (2024)
I/II	Gadolinium-based Nanocarriers	Glioblastoma	47	OS, ORR, MTD, DLT	Combination therapy with AGuIX nanocarriers improved therapeutic outcomes in newly diagnosed glioblastoma patients.	Addressing inter-patient variability in nanocarrier distribution and radiosensitivity.	Thivat et al. (2023)

Abbreviations: CI, Cardiotoxicity Incidence; DLT, Dose-Limiting Toxicity; GSE, Gene Silencing Duration; MTD, Maximum Tolerated Dose; ORR, Objective Response Rate; OS, Overall Survival; PFS, Progression-Free Survival; TE, tumor accumulation efficiency.

Importantly, cardio-oncology nanomedicine distinguishes itself through its fundamental dual-targeting paradigm. By simultaneously enabling tumor-specific drug delivery and controlled release of cardioprotective agents, it addresses a long-standing challenge in oncology: enhancing anticancer efficacy while actively safeguarding cardiac function. This integrative approach elevates cardio-oncology nanomedicine as a distinct and emerging discipline within the broader field of precision oncology.

## 5 Innovation and future prospects

### 5.1 Development of nanotechnology integrated with artificial intelligence

The convergence of nanotechnology with artificial intelligence (AI) and machine learning (ML) is opening new frontiers for the design of next-generation nanocarriers in oncology (Tan et al., 2023). AI enables the analysis of large and complex biological datasets, facilitating the development of nanocarriers with enhanced specificity and reduced toxicity (Corti et al., 2023). For example, Chou et al. used an AI-assisted pharmacokinetic model to optimize nanocarrier size, surface chemistry, and dosing for targeted tumor delivery (Chou et al., 2023), while Zhang et al. applied machine learning to rapidly screen functional nanomedicines via drug-drug self-assembly (Zhang et al., 2025). Furthermore, real-time AI-driven monitoring systems can guide individualized dose adjustments according to patient responses, thereby improving therapeutic precision and outcomes (Bhinder et al., 2021; Pang et al., 2022). With continued advances, AI is expected to transform precision medicine by accelerating nanocarrier design and enabling more efficient, tumor-targeted interventions.

### 5.2 Personalized nanomedicine delivery

The rise of personalized medicine has intensified interest in patient-specific nanocarrier systems. Personalized nanomedicine leverages molecular and biological markers to optimize therapeutic efficacy (Passaro et al., 2024). By incorporating factors such as gene expression patterns, protein profiles, and metabolic signatures, nanocarriers can be tailored to improve drug delivery precision and clinical outcomes (Zhou et al., 2024). This approach is particularly valuable for addressing tumor heterogeneity and patient-to-patient variability in treatment response. For example, targeting receptors that are overexpressed in specific cancers, such as HER2 in breast cancer, enables direct delivery of chemotherapeutic agents to malignant cells while minimizing systemic toxicity (Krishnamurti and Silverman, 2014; Ratajczak et al., 2023). Ongoing progress in genomics and proteomics is accelerating the development of customized nanocarrier formulations aligned with each patient’s genetic and molecular landscape, positioning personalized nanomedicine as a central component of future cancer therapy.

### 5.3 Integration of multifunctional nanotechnology

**FIGURE 7:**
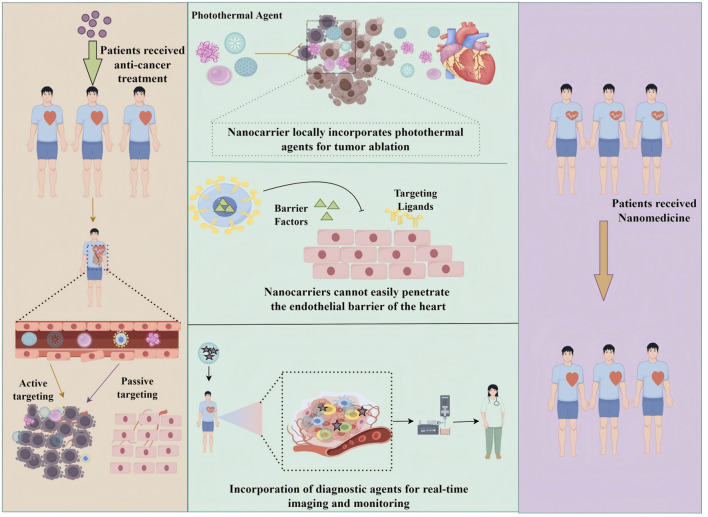
Schematic representation of multifunctional nanocarriers for simultaneous drug delivery, imaging, and cardiac protection.

A key future direction in cancer therapy lies in the integration of multifunctional nanotechnology with diverse therapeutic modalities. Multifunctional nanoplatforms can simultaneously combine chemotherapy with photothermal therapy, immunotherapy, or gene therapy, thereby enhancing therapeutic efficacy (Ashrafizadeh et al., 2023; Kang et al., 2023; Overchuk et al., 2023). For example, nanocarriers engineered to deliver both chemotherapeutics and immune checkpoint inhibitors can potentiate antitumor immune responses (Liang et al., 2024). The incorporation of photothermal agents into nanocarriers enables the concurrent release of drugs and localized hyperthermia, which increases tumor cell susceptibility to treatment (Dorjsuren et al., 2020). Moreover, nanocarriers are being developed as vehicles for gene therapy, enabling the correction of tumor-specific genetic alterations (Yu et al., 2021). Figure 7 illustrates multifunctional nanocarriers that integrate drug delivery, imaging, and cardio-protection within a single system, underscoring their potential to achieve multiple therapeutic objectives concurrently. Such multifunctional strategies represent a transformative shift in oncology, where a single nanoplatform can synergistically combine several treatment modalities, offering a comprehensive and highly effective approach to combating cancer.

## 6 Discussion and conclusion

Central to this review is the paradigm-shifting concept of cardio-oncology nanotechnology, which is defined by its dual commitment to antitumor efficacy and cardio-protection. This duality distinguishes it from conventional nanomedicine approaches that focus exclusively on tumor targeting (Lu et al., 2024). The analysis presented here highlights the transformative role of nanotechnology in cancer therapy, particularly in addressing CIC while maintaining robust antitumor activity. Nanocarriers such as liposomes, polymeric nanocarriers, and inorganic nanomaterials enhance the precision of drug delivery through both passive and active targeting mechanisms (Garbayo et al., 2020; Vazhappilly et al., 2021). More importantly, these platforms establish a novel therapeutic paradigm by integrating tumor suppression with active cardio-protection, a synergistic framework that defines the innovation of this emerging discipline (Yu et al., 2021). Recent advances in cardioprotective nanocarriers have reduced off-target effects and mitigated cardiac injury, while preclinical and clinical studies have demonstrated encouraging improvements in patient outcomes (Rafiyath et al., 2012; Keefe et al., 2016; Yang et al., 2022). Collectively, these findings establish cancer nanocardiology as a distinct research ecosystem characterized by standardized models for evaluating integrated therapeutic and protective efficacy. This dual-functional strategy underscores the capacity of nanotechnology to render cancer treatments both safer and more effective, while also pointing toward future developments in artificial intelligence-driven optimization and personalized medicine.

The findings of this review support prior evidence that nanocarrier-based drug delivery significantly reduces systemic damage compared to conventional formulations (Nooreen et al., 2022; Zhang L. et al., 2023; Alarcon et al., 2025). For example, liposomal DOX consistently reduces CIC by up to 54%, as reported in multiple studies and meta-analyses (Xing et al., 2015). However, this review extends current knowledge by emphasizing the incorporation of cardioprotective agents into nanocarriers, an underexplored yet promising strategy (Bruno et al., 2021; Kong et al., 2022). Additionally, the increasing use of pH-sensitive and multi-stimuli-responsive nanocarriers offers new opportunities to enhance therapeutic precision (Liu et al., 2014; Kong et al., 2023). By embedding cardioprotection into the broader framework of oncological nanomedicine, this review addresses critical gaps that remain in the field.

Despite these advances, several barriers limit the widespread clinical translation of nanocarrier systems. First, the variability of tumor microenvironments constrains the effectiveness of passive targeting strategies such as the EPR effect (Izci et al., 2021; Yang et al., 2021). Second, the long-term effects of nanocarriers, including their potential immunomodulatory properties and accumulation in tissues, remain insufficiently understood (Saifi et al., 2021; Zhao et al., 2022). Third, challenges in scaling up production and the high cost of manufacturing multifunctional nanocarriers pose significant practical obstacles (Pang et al., 2023). These limitations highlight the need for further optimization and rigorous evaluation of nanocarrier systems in experimental and clinical settings.

Thus, future innovation must refine the dual-functional architecture of nanocarriers, with AI serving as a key enabler for improving spatiotemporal precision in balancing tumor suppression and cardioprotection (Li et al., 2025). Machine learning approaches can facilitate predictive modeling of tumor characteristics, enabling the customization of nanocarrier properties such as size, charge, and surface chemistry (Chen, 2023). Furthermore, the development of recyclable or bio-derived nanocarriers may address concerns regarding the long-term health and environmental impacts of synthetic nanomaterials (Umapathi et al., 2022). Combining nanotechnology with gene therapy and immune-based strategies also presents considerable promise for expanding therapeutic capabilities (Kiaie et al., 2023; Birnboim-Perach and Benhar, 2024). Ultimately, large-scale, rigorously designed clinical trials remain essential for validating the safety, efficacy, and cost-effectiveness of nanocarriers, thereby enabling broader clinical adoption (Su et al., 2022; Saadh et al., 2024).

In summary, this review underscores the transformative potential of nanotechnology in cancer treatment, demonstrating its ability to enhance therapeutic efficacy while minimizing cardiotoxicity. Beyond oncology, the principles of dual-functional nanomedicine may serve as a model for other areas, including regenerative medicine and infectious disease management, underscoring the broad societal relevance of this field (Zhang P. et al., 2023; Abu Elella and Kolawole, 2024).
